# Diversity and distribution of the millipedes (Diplopoda) of Georgia, Caucasus

**DOI:** 10.3897/zookeys.930.47490

**Published:** 2020-04-28

**Authors:** Mzia S. Kokhia, Sergei I. Golovatch

**Affiliations:** 1 Institute of Zoology, Ilia State University, K. Cholokashvili Ave., 3/5, Tbilisi 0162, Georgia Ilia State University Tbilisi Georgia; 2 Institute for Problems of Ecology and Evolution, Russian Academy of Sciences, Leninsky prospekt 33, Moscow 119071, Russia Institute for Problems of Ecology and Evolution, Russian Academy of Sciences Moscow Russia

**Keywords:** checklist, Colchis, endemism, fauna, Myriapoda

## Abstract

The diplopod fauna of Georgia, Transcaucasia, is very rich given the country’s relatively small territory; it presently comprises 103 species from 44 genera, 12 families, and 7 orders. Most of the Diplopoda known from Georgia (86 species, or 83%) demonstrate Caucasian distribution patterns, 36 and 46 species, as well as 8 and 9 genera being endemic or subendemic to the country, respectively. A single Holarctic family, Anthroleucosomatidae (order Chordeumatida), contains 44 Caucasian species and 20 genera, of which 27 species and 14 genera are endemic or subendemic to Georgia. Likewise, all species from the orders Polyzoniida, Siphonocryptida, Glomerida and Chordeumatida, as well as most species of Julida and Polydesmida are native, also endemic or subendemic to the Caucasus, but the genera and families they represent are widely distributed at least across the Euro-Mediterranean Realm. Most of the presumed troglobionts in the Caucasus appear to be confined to western Georgia’s karst caves (14 species, 5 genera). Within Georgia, the fauna of the western part (= Colchis) is particularly rich and diverse, while that of the central and eastern parts of the country grows increasingly depauperate inland following the gradual climatic aridisation from west (Black Sea coast) to east (Armenia and Azerbaijan). The vertical distribution of the Diplopoda in Georgia, as well as the Caucasus generally, shows the bulk of the fauna restricted to forested lowland to mountain biomes or their remnants. Only very few Chordeumatida and *Julus* species seem to occur solely in the subalpine to alpine environments and thus may provisionally be considered as high-montane elements. Ongoing and future research on the millipedes of the Caucasus, especially in cave and montane environments, will undoubtedly allow for many more novelties and details of the diversity and distribution of Georgia’s Diplopoda to be revealed or refined.

## Introduction

**Figure 1.**
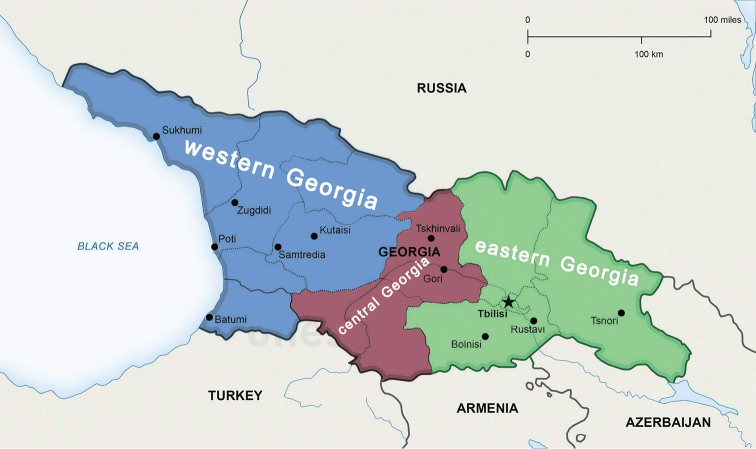
Geographic division of Georgia.

Georgia is one of the main countries in the Caucasus, lying between western Asia and Eastern Europe. It is bounded in the west by the Black Sea, in the north by Russia, in the south by Turkey, and in the southeast and east by Armenia and Azerbaijan (Fig. 1). The area is mainly montane to high montane, situated between 41° and 44°N, and 40° and 47°E. The Greater Caucasus Mountain Range, or Caucasus Major, forms the northern border of Georgia, while the southern border is bounded by the Lesser Caucasus Mountains, or Caucasus Minor. The Caucasus Major is much higher in elevation (more than 5000 m a.s.l.) than the plateau-like Caucasus Minor, both being connected by the submeridional Surami (= Likhi) Mountain Range which divides Georgia into the western and central + eastern parts. Both parts are quite varied in climate and biota. Western Georgia’s landscape ranges from lowland marsh-forests, swamps, and temperate rainforests within the Colchis Plain to eternal snows and glaciers, while the eastern part of the country even contains a small segment of semi-arid plains. Forests cover around 40% of Georgia’s territory, while the alpine/subalpine zone accounts for approximately 10% of the land. The climate of Georgia is extremely diverse, considering the nation’s small size, but is largely mild to warm. There are two main climatic zones, roughly corresponding to the eastern and western parts of the country. The Greater Caucasus Mountain Range plays an important role in moderating Georgia’s climate and protects the nation from the penetration of colder air masses from the north. The Lesser Caucasus Mountains partially protect the region from the influence of dry and hot air masses from the south (Bondyrev et al. 2015).

The millipede fauna of Georgia has recently been reviewed and shown to comprise 95 species from 42 genera, 12 families, and 7 orders (Kokhia and Golovatch 2018). A few relevant faunistic papers have, or will have, appeared since (Golovatch 2018, Golovatch and Turbanov 2017, Antić et al. 2018, Evsyukov et al. 2018, 2020, Vagalinski and Lazányi 2018, Short et al. 2020), allowing for the previous checklist to be rectified and updated, as well as the previous reference list to be considerably shortened. The present checklist contains 103 species from 44 genera, 12 families, and 7 orders (Table 1). Data on the elevations at which the species occur, both within and beyond Georgia, are also added, representing the basic information for our analysis of millipede vertical distributions.

## Material and methods

Only described species and published records are considered in our paper, while dubious taxa and those not identified to the species level have been omitted both from the checklist and reference list. Only one important exception has been made: *Calyptophyllum* sp. as the only record of this genus in the Caucasus (Table 1).

**Figure 2.**
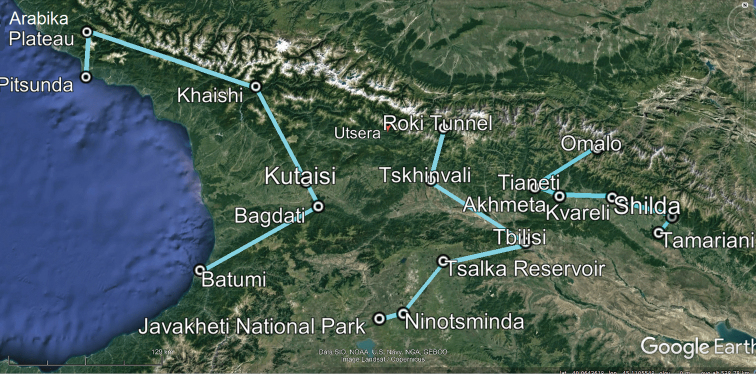
Map of Georgia with three transects (light blue), one each in the western, central and eastern parts of the country, to crudely show both horizontal and vertical distributions of millipedes endemic or subendemic to the country.

**Figure 3.**
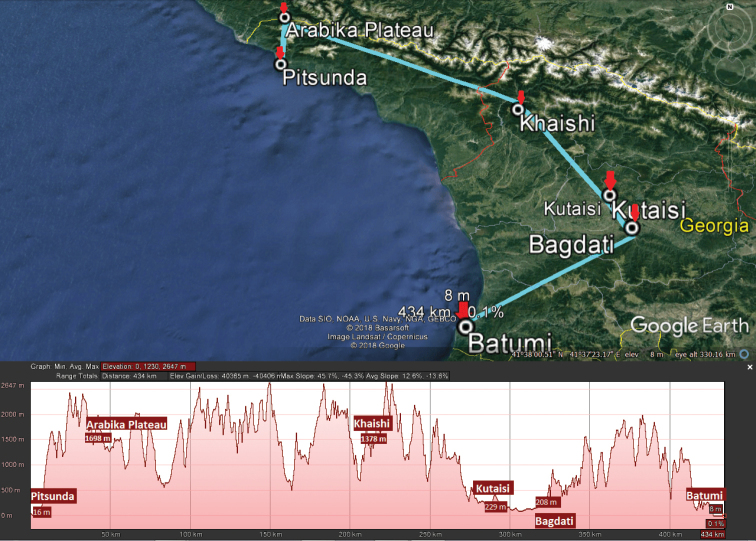
Map of western Georgia with its transect (light blue), Pitsunda – Arabika Plateau – Khaishi – Bagdati – Batumi, and macro relief (bottom).

**Figure 4.**
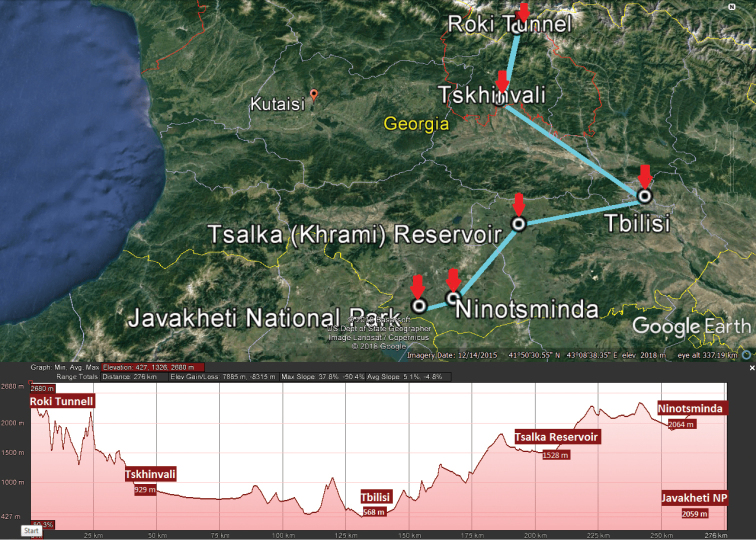
Map of central Georgia with its transect (light blue), Roki Tunnel – Tskhinvali – Tbilisi – Tsalka Reservoir – Ninotsminda – Javakheti National Park, and its macro relief (bottom).

**Figure 5.**
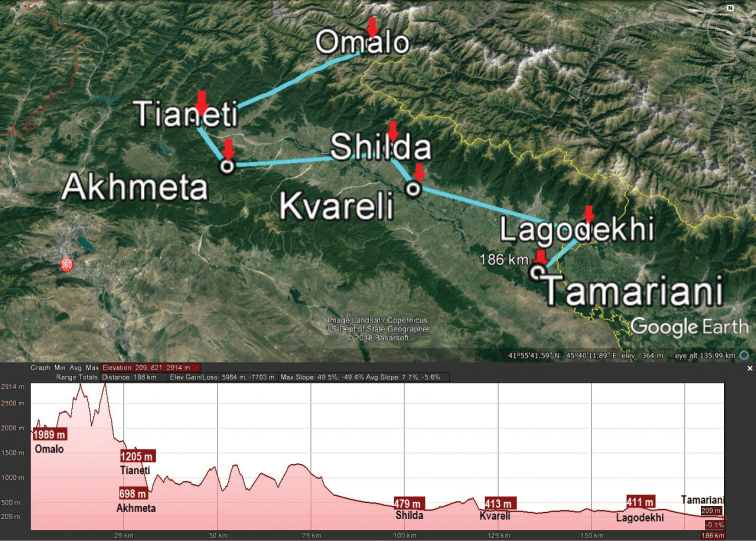
Map of eastern Georgia with its transect (light blue), Omalo – Tianeti – Akhmeta – Shilda – Kvareli – Lagodekhi – Tamariani, and its macro relief (bottom).

Three zigzag transects chosen to grossly reflect the north-to-south lie of the macro relief of Georgia, extending from the Caucasus Major in the north to the Caucasus Minor in the south (Figs 2–5), have been drawn, one each for the western, central and eastern parts of the country (Fig. 1). The transect across western Georgia connects Pitsunda – Arabika Plateau – Khaishi – Bagdati – Batumi (427 km long), that in central Georgia connects Roki Tunnel – Tskhinvali – Tbilisi – Tsalka Reservoir – Ninotsminda – Javakheti National Park (275 km), and the eastern Georgia one connects Omalo – Tianeti – Akhmeta – Shilda – Kvareli – Lagodekhi – Tamariani (186 km) (Fig. 2). Both at the bottom of the maps and on the maps themselves, each transect is accompanied by the respective altitudes given for each of the turn localities and thus provides a clear generalized picture of the macro relief (Figs 3–5). These three transects thus cover all major variations in millipede vertical distribution across entire Georgia. This novel approach to a graphic presentation of faunistic results allows us to combine the horizontal and vertical distributions of millipedes in the easiest and most vivid way on the same map. Mapping largely concerns endemic or subendemic species and concerns only the territory of Georgia.

Most of the colour maps were generated using Google Earth Pro version 7.3.2.5495 and Adobe Photoshop CS6. The final images were processed with Adobe Photoshop CS6.

## Results

The diplopod fauna of the Caucasus region, including Georgia, is basically Euro-Mediterranean in its composition (Table 1). This also concerns the relatively few widespread, likely introduced species from the orders Polyxenida, Julida and Polydesmida that occur in the Caucasus. Even among the few unquestioned introductions, only *Oxidus gracilis* (C.L. Koch, 1847) is an Oriental or East Asian alien element.

**Table 1.** A revised checklist of the Diplopoda of Georgia, with data on species distributions, both within and beyond the country, their statuses, and the main relevant literature sources. Designations: i – introduced; G – entire Georgia; W – western Georgia; C – central Georgia; E – eastern Georgia; R – Russian Caucasus; T – Turkey; Ar – Armenia; Az – Azerbaijan; Cr – Crimean Peninsula; (+) – present; e – endemic to Georgia; se – subendemic to Georgia; t – presumed troglobiont; sc – subcosmopolitan; EuM – Euro-Mediterranean; M – Mediterranean; EM – eastern Mediterranean; EE – eastern European; Ca – Caucasian.

Fauna	G	R	T	Ar	Az	Cr	Elevations (m a.s.l.) and status	Distribution pattern	Main relevant references
Class Diplopoda									
Order Polyxenida									
Family Polyxenidae									
Genus *Polyxenus* Latreille, 1803									
1. *Polyxenus lagurus* (Linnaeus, 1758)	W	+				+	20–1700, i	sc	Issaev 1911, Short et al. 2020
2. *Polyxenus lankaranensis* Short, Vahtera, Wesener & Golovatch, 2020	E	+			+		100–800	Ca	Short et al. 2020
Genus *Propolyxenus* Silvestri, 1948	W								
3. *Propolyxenus argentifer* (Verhoeff, 1921)	G	+	+	+	+	+	20–1700	EM	Short et al. 2020
Family Lophoproctidae									
Genus *Lophoproctus* Pocock, 1894									
4. *Lophoproctus coecus* Pocock, 1894	G					+	20–900	EM	Short 2015, Short et al. 2020
Order Polyzoniida									
Family Hirudisomatidae									
Genus *Hirudisoma* Fanzago, 1881									
5. *Hirudisoma roseum* (Victor, 1839)	G	+	+		+		20–1100, se	EM	Golovatch et al. 2015
Order Siphonocryptida									
Family Siphonocryptidae									
*Hirudicryptus* Enghoff & Golovatch, 1985									
6. *Hirudicryptus abchasicus* Golovatch, Evsyukov & Reip, 2015	W	+					600–1500, se	Ca	Golovatch et al. 2015, Zuev 2017
Order Glomerida									
Family Glomeridae									
Genus *Hyleoglomeris* Verhoeff, 1910									
7. *Hyleoglomeris awchasica* (Brandt, 1840)	W	+					20–2100, se	Ca	Golovatch 1975, 1976a, 1989b
8. *H. specialis* Golovatch, 1989	E	+					500–1400, se	Ca	Golovatch 1989b
Genus *Trachysphaera* Heller, 1858									
9. *Trachyspaera costata* (Waga, 1857)	G	+	+	+	+	+	20–2000	EuM	Golovatch 1990, 2008
10. *T. fragilis* Golovatch, 1976	G	+					80–460, t, e	Ca	Golovatch 1976c,^1990^, Golovatch and Turbanov 2017
11. *T. minuta* Golovatch, 1976	G	+	+	+			20–1700, se	Ca	Golovatch 1976c, 1990
12. *T. orientalis* Golovatch, 1976	W						800–1100, t, e	Ca	Golovatch 1976c, 1990
13. *T. radiosa* (Lignau, 1911)	W	+					20–1800, se	Ca	Golovatch 1976a, 1990
14. *T. solida* Golovatch, 1976	W, C						20–2020, se	Ca	Golovatch 1976c, 1976c, 1990, 1993
Family Glomeridellidae									
Genus *Typhloglomeris* Verhoeff, 1898									
15. *Typhloglomeris lohmanderi* (Golovatch, 1989)	C, E	+		+			600–1450, se	Ca	Golovatch 1989a, 2003
16. *Typhloglomeris palatovi* Golovatch & Turbanov, 2018	W						650, t, e	Ca	Golovatch and Turbanov 2018
Order Chordeumatida									
Family Anthroleucosomatidae									
Genus *Acanthophorella* Antić & Makarov, 2016									
17. *Acanthophorella barjadzei* Antić & Makarov, 2016	W						1120–1200, t, e	Ca	Antić and Makarov 2016
Genus *Adshardicus* Golovatch, 1981									
18. *Adshardicus strasseri* Golovatch, 1981	W		+				20–530, se	Ca	Enghoff 2006, Antić and Makarov 2016
Genus *Alpinella* Antić & Makarov, 2016									
19. *Alpinella waltheri* Antić & Makarov, 2016	E						2860, e	Ca	Antić and Makarov 2016
Genus *Brachychaetosoma* Antić & Makarov, 2016									
20. *Brachychaetosoma turbanovi* Antić & Makarov, 2016	W						300, t, e	Ca	Antić and Makarov 2016
Genus *Caucaseuma* Strasser, 1970									
21. *Caucaseuma kelasuri* Antić & Makarov, 2016	W						190, e	Ca	Antić and Makarov 2016
22. *C. variabile* Antić & Makarov, 2016	C	+					100–2500, se	Ca	Antić and Makarov 2016
Genus *Cryptacanthophorella* Antić & Makarov, 2016									
23. *Cryptacanthophorella manubriata* Antić & Makarov, 2016	W, C						800–1700, e	Ca	Antić and Makarov 2016
Genus *Dentatosoma* Antić & Makarov, 2016									
24. *Dentatosoma denticulatum* Antić & Makarov, 2016	W						400–900, e	Ca	Antić and Makarov 2016
25. *D. magnum* Antić & Makarov, 2016	W	+					20–2200, se	Ca	Antić and Makarov 2016
26. *D. zeraboseli* Antić & Makarov, 2016	W						20–1700, e	Ca	Antić and Makarov 2016
Genus *Georgiosoma* Antić & Makarov, 2016									
27. *Georgiosoma bicornutum* Antić & Makarov, 2016	W						2000, t, e	Ca	Antić and Makarov 2016
Genus *Herculina* Antić & Makarov, 2016									
28. *Herculina oligosagittae* Antić & Makarov, 2016	W						1500–1700, e	Ca	Antić and Makarov 2016
29. *H. polysagittae* Antić & Makarov, 2016	C						1750, e	Ca	Antić and Makarov 2016
Genus *Heterocaucaseuma* Antić & Makarov, 2016									
30. *Heterocaucaseuma deprofundum* Antić & Makarov, 2018	W						2000–2100, t, e	Ca	Antić et al. 2018
31. *H. longicorne* Antić & Makarov, 2016	W						100–350, t, e	Ca	Antić and Makarov 2016, Antić et al. 2018
32. *H. mauriesi* (Golovatch & Makarov, 2011)	W						215, t, e	Ca	Golovatch and Makarov 2011, Antić and Makarov 2016, Antić et al. 2018
Genus *Metamastigophorophyllon* Ceuca, 1976									
33. *Metamastigophorophyllon giljarovi* (Lang, 1959)	W	+					20–1850, se	Ca	Antić and Makarov 2016
34. *M. hamatum* Antić & Makarov, 2016	W	+					150–2200, se	Ca	Antić and Makarov 2016
35. *M. lamellohirsutum* Antić & Makarov, 2016	W						700–800, e	Ca	Antić and Makarov 2016
36. *M. torsivum* Antić & Makarov, 2016	G				+		800–1700, se	Ca	Antić and Makarov 2016
Genus *Paranotosoma* Antić & Makarov, 2016									
37. *Paranotosoma attemsi* Antić & Makarov, 2016	W						1500–1800, e	Ca	Antić and Makarov 2016
38. *P. cordatum* Antić & Makarov, 2016	W						20–800, e	Ca	Antić and Makarov 2016
39. *P. subrotundatum* Antić & Makarov, 2016	W, C	+					350–850, se	Ca	Antić and Makarov 2016
Genus *Pseudoflagellophorella* Antić & Makarov, 2016									
40. *Pseudoflagellophorella eskovi* Antić & Makarov, 2016	C, E			+	+		100–2080, se	Ca	Antić and Makarov 2016
41. *P. mirabilis* Antić & Makarov, 2016	W						20–130, e	Ca	Antić and Makarov 2016
42. *P. papilioformis* Antić & Makarov, 2016	E				+		850–2100, se	Ca	Antić and Makarov 2016
Genus *Ratcheuma* Golovatch, 1985									
43. *Ratcheuma excorne* Golovatch, 1985	W						1180, t, e	Ca	Golovatch 1984/85, Antić and Makarov 2016
Order Julida									
Family Blaniulidae									
Genus *Cibiniulus* Verhoeff, 1927									
44. *Cibiniulus phlepsii* (Verhoeff, 1897)	W		+				20–130	EuM	Enghoff 1984, 2006
Genus *Nopoiulus* Menge, 1851									
45. *Nopoiulus brevipilosus* Enghoff, 1984	W						130, t, e	Ca	Enghoff 1984, Golovatch and Enghoff 1990
46. *N. densepilosus* Enghoff, 1984	W		+		+		1500–1700	Ca	Enghoff 1984, Golovatch and Enghoff 1990
47. *N. golovatchi* Enghoff, 1984	W		+				20–130, se	Ca	Enghoff 1984, 1990
48. *N. kochii* (Gervais, 1847)	G	+	+	+	+		10–2200, i?	sc	Enghoff 1984, Golovatch and Enghoff 1990
Family Nemasomatidae									
Genus *Nemasoma* C.L. Koch, 1847									
49. *Nemasoma caucasicum* (Lohmander, 1932)	G	+	+	+	+		20–2000, se	Ca	Kobakhidze 1965, Enghoff 1985
Family Julidae									
Genus *Archileucogeorgia* Lohmander, 1936									
50. *Archileucogeorgia abchasica* Lohmander, 1936	W						130, t, e	Ca	Lohmander 1936
51. *A. satunini* Lohmander, 1936	W						130, e	Ca	Lohmander 1936
Genus *Brachyiulus* Berlese, 1884									
52. *Brachyiulus lusitanus* Verhoeff, 1898`	C				+		100, i	M	Lohmander 1936
Genus *Byzantorhopalum* Verhoeff, 1930									
53. *Byzantorhopalum rossicum* (Timotheew, 1897)	W?	+			+	+	30–1500	EE	Lohmander 1936, Vagalinski and Lazányi 2018
Genus *Catamicrophyllum* Verhoeff, 1900									
54. *Catamicrophyllum caucasicum* (Attems, 1901)	G	+	+	+			700–2000, se	Ca	Lohmander 1936, Enghoff 1995
Genus *Calyptophyllum* Brolemann, 1922									
55. *Calyptophyllum* sp.	W						100?	?	Lohmander 1936, Enghoff 1995
Genus *Chaetoleptophyllum* Verhoeff, 1898									
56. *Chaetoleptophyllum flexum* Golovatch, 1979	G	+					15–2200, se	Ca	Golovatch 1979, Evsyukov et al. (2020)
Genus *Cylindroiulus* Verhoeff, 1894									
57. *Cylindroiulus bellus* (Lignau, 1903)	W?	+				+	100	EM	Lignau 1903, Read 1992, Chumachenko 2016
58. *C. crassiphylacum* Read, 1992	W, C		+				600–1700, se	Ca	Read 1992
59. *C. kacheticus* Lohmander, 1936	E	+					500–1250, se	Ca	Lohmander 1936, Read 1992
60. *C. olgainna* Read, 1992	W						300–1100, e	Ca	Read 1992
61. *C. parvus* Lohmander, 1928	C, E				+		500–2100, se	Ca	Lohmander 1936, Read 1992
62. *C. placidus* (Lignau, 1903)	W, C	+					20–2200, se	Ca	Lignau 1903, Read 1992
63. *C. pterophylacum* Read, 1992	W, C	+					20–1600, se	Ca	Read 1992, Zuev 2014
64. *C. quadrus* Read, 1992	W, C						700–1000, e	Ca	Read 1992
65. *C. ruber* (Lignau, 1903)	W	+					100–2000, se	Ca	Lignau 1903, 1915, Read 1992
66. *C. schestoperovi* Lohmander, 1936	W	+					400–1800, se	Ca	Lohmander 1936, Read 1992
67. *C. truncorum* (Silvestri, 1896)	W	+	+				130, i	sc	Read 1992
Genus *Grusiniulus* Lohmander, 1936									
68. *Grusiniulus redikorzevi* Lohmander, 1936	C						800–900, e	Ca	Lohmander 1936, Vagalinski and Lazányi 2018
Genus *Julus* Linnaeus, 1758									
69. *Julus colchicus* Lohmander, 1936	G	+	+				20–2850, se	Ca	Lohmander 1936, Enghoff 2006, Evsyukov et al. 2018
70. *J. kubanus* Lohmander, 1936	W, E	+					300–2100, se	Ca	Lohmander 1936, Kobakhidze 1965, Evsyukov et al. 2018
71. *J. lignaui* Verhoeff, 1910	W	+					1500–2800, se	Ca	Evsyukov et al. 2018
72. *J. lindholmi* Lohmander, 1936	W	+					450–2200, se	Ca	Lohmander 1936, Evsyukov et al. 2018
Genus *Kubaniulus* Lohmander, 1936									
73. *Kubaniulus gracilis* Lohmander, 1936	W	+					20–700, se	Ca	Lohmander 1936, Evsyukov et al. 2020
Genus *Leptoiulus* Verhoeff, 1894									
74. *Leptoiulus hastatus* Lohmander, 1932	C		+				800–1530, se	Ca	Lohmander 1936, Enghoff 2006, Evsyukov et al. 2020
75. *L. tanymorphus* (Attems, 1901)	C, E	+		+	+		80–1800, se	Ca	Lohmander 1936, Evsyukov et al. 2020
Genus *Leucogeorgia* Verhoeff, 1930									
76. *Leucogeorgia longipes* Verhoeff, 1930	W						170, t, e	Ca	Verhoeff 1930, Barjadze et al. 2019
77. *L. rediviva* Golovatch, 1983	W						330, t, e	Ca	Golovatch 1983, Barjadze et al. 2019
Genus *Megaphyllum* Verhoeff, 1894									
78. *Megaphyllum dioscoriadis* (Lignau, 1915)	W	+					130–1400, se	Ca	Lignau 1915, Lohmander 1936, Kobakhidze 1965, Chumachenko 2016, Vagalinski and Lazányi 2018
79. *M. hercules* (Verhoeff, 1901)	W	+					20, i	EM	Lazányi and Vagalinski 2013
80. *M. spathulatum* (Lohmander, 1936)	W?	?					?	Ca	Lohmander 1936, Lazányi and Vagalinski 2013
Genus *Omobrachyiulus* Lohmander, 1936									
81. *Omobrachyiulus adsharicus* (Lohmander, 1936)	W						20–30, e	Ca	Lohmander 1936, Vagalinski and Lazányi 2018
82. *O. brachyurus* (Attems, 1899)	G	+	+	+	+		20–2500	EM	Lohmander 1936, Kobakhidze 1965, Enghoff 2006, Vagalinski and Lazányi 2018
83. *O. curvocaudatus* (Lignau, 1903)	W	+					30–1700, se	Ca	Lohmander 1936, Kobakhidze 1965, Vagalinski and Lazányi 2018
84. *O. divaricatus* (Lohmander, 1936)	G			+			600 –2000, se	Ca	Lohmander 1936, Kobakhidze 1965, Vagalinski and Lazányi 2018
85. *O. hortensis* (Golovatch, 1981)	W						150, e	Ca	Golovatch 1981, Vagalinski and Lazányi 2018
86. *O. implicitus* Lohmander, 1936 (= *O. i. ritsensis* (Golovatch, 1981))	W	+					400–1800, se	Ca	Lohmander 1936, Chumachenko 2016, Vagalinski and Lazányi 2018, Vagalinski in litt.
87. *O. macrourus* (Lohmander, 1928) (= *O. m. abchasicus* (Lohmander, 1936))	W, C						130–2000, e	Ca	Lohmander 1936, Kobakhidze 1965, Vagalinski and Lazányi 2018, Vagalinski in litt.
Genus *Pachyiulus* Berlese, 1883									
88. *Pachyiulus flavipes* (C.L. Koch, 1847)	W					+	30, i	M	Lohmander 1936
89. *P. krivolutskyi* Golovatch, 1977	W	+					20–1800, se	Ca	Golovatch 1977, Evsyukov 2016
Genus *Syrioiulus* Verhoeff, 1914									
90. *Syrioiulus adsharicus* (Lohmander, 1936)	W						120, e	Ca	Lohmander 1936, Golovatch 2018
91. *S. georgicus* (Lohmander, 1932)	C						800–900, e	Ca	Lohmander 1932, Golovatch, 2018
Order Polydesmida									
Family Paradoxosomatidae									
Genus *Oxidus* Cook, 1911									
92. *Oxidus gracilis* (C.L. Koch, 1847)	W	+					20–100, i	Ca	Lignau 1915, Lohmander 1936, Chumachenko 2016
Genus *Strongylosoma* Brandt, 1833									
93. *Strongylosoma kordylamythrum* Attems, 1898	G	+		+			20–2200	Ca	Lohmander 1936, Kobakhidze 1965, Evyukov et al. 2016
94. *S. lenkoranum* Attems, 1898	C		+	+	+		80–1650	Ca	Lohmander 1936, Kobakhidze 1965, Evyukov et al. 2016
Family Polydesmidae									
Genus *Brachydesmus* Heller, 1858									
95. *Brachydesmus assimilis* Lohmander, 1936	C, E	+		+	+		600–2800, se	Ca	Golovatch et al. 2016
96. *B. furcatus* Lohmander, 1936	W	+					20–1900, se	Ca	Golovatch et al. 2016
97. *B. kalischewskyi* Lignau, 1915	G	+	+	+	+		50–2400, se	Ca	Golovatch et al. 2016
98. *B. kvavadzei* Golovatch, Evsyukov & Reip, 2016	W						70–1520, e	Ca	Golovatch et al. 2016
99. *B. simplex* Golovatch, Evsyukov & Reip, 2016	W	+					20–1100, se	Ca	Golovatch et al. 2016
100. *B. superus* Latzel, 1884	W	+					150–450, i	sc	Golovatch et al. 2016
Genus *Polydesmus* Latreille, 1803									
101. *Polydesmus abchasius* Attems, 1899	W, C	+					10–2230, se	Ca	Golovatch et al. 2016
102. *P. lignaui* Lohmander, 1936	W	+					100–2200, se	Ca	Golovatch et al. 2016
103. *P. mediterraneus* Daday, 1889	W					+	100, i	EM	Golovatch et al. 2016

All species of Polyzoniida, Siphonocryptida, Glomerida and Chordeumatida, as well as most species of Julida and Polydesmida appear to be native, endemic or subendemic, but the genera and families they represent are widely distributed across the Euro-Mediterranean Realm. As a result, endemism is profound at the species and, to a lesser degree, generic levels. Most of the species (86, or 83%) show a Caucasian distribution pattern, thus being endemic or subendemic to the Caucasus region. The same pattern was found at the generic level, with 18 genera being endemic or subendemic to the Caucasus, including all 14 genera of the order Chordeumatida that inhabit the region (Antić and Makarov 2016, Antić et al. 2018). There are neither families nor orders of Diplopoda that are confined to the Caucasus region alone.

Our analysis of the distribution of Georgia’s millipedes is largely based on strictly endemic and subendemic species (36 and 46, respectively: Table 1) and genera (8 and 9, respectively: *Alpinella*, *Brachychaetosoma*, *Cryptacanthophorella*, *Georgiosoma*, *Grusiniulus. Herculina*, *Leucogeorgia* and *Ratcheuma*, vs. *Adshardicus*, *Acanthophorella*, *Archileucogeorgia*, *Caucaseuma*, *Dentatosoma*, *Heterocaucaseuma*, *Omobrachyiulus*, *Paranotosoma* and *Pseudoflagellophorella*). It shows that western Georgia, including Abkhazia and Ajaria – which are shown separately (Figs 9, 10) to more clearly depict the localities/distributions and thus to avoid an “overcrowded” picture – supports the richest and most diverse fauna (Figs 7–10). This is also the area where all 14 presumed troglobionts are found in Georgia, all confined to karst caves (Barjadze et al. 2019). Abkhazia, northwestern Georgia, is the richest subregion both in epigean and troglobitic Diplopoda (Figs 7–9), hosting, among others, *Heterocaucaseuma deprofundum* Antić & Makarov, 2018. This species is the world’s deepest record of a millipede, found at 60–1980 m below the surface in the Krubera-Voronja and Sarma caves, Arabika Massif, Abkhazia (Fig. 3). Both these caves are among the deepest globally and support the second and third deepest subterranean invertebrate communities, respectively. Furthermore, both harbour still one more diplopod species, a yet undescribed *Leucogeorgia* sp. (Antić et al. 2018).

**Figure 6.**
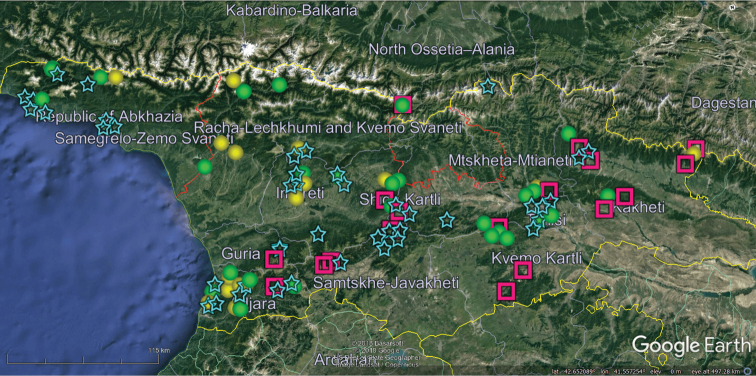
Map showing the distributions of four particularly widespread millipedes endemic or subendemic to Georgia. Designations: yellow ball (*Hirudisoma roseum*), green ball (*Chaetoleptophyllum flexum*), pink square (*Metamastigophorophyllon torsivum*), blue star (*Julus colchicus*).

**Figure 7.**
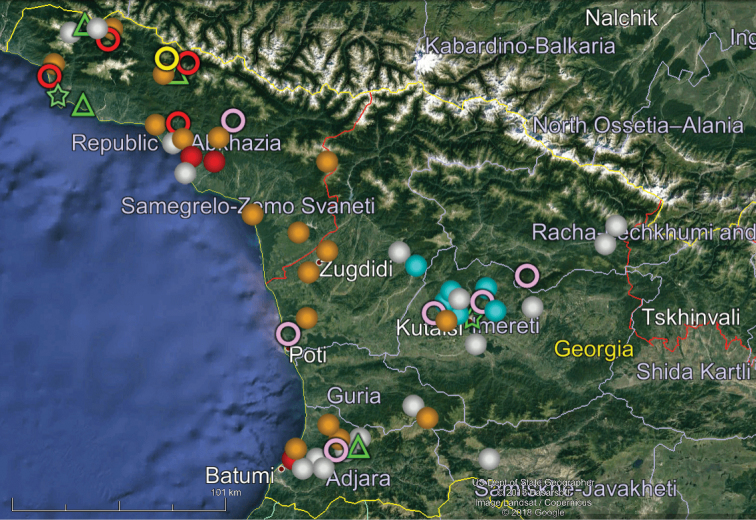
Map of western Georgia (= Colchis) showing the distributions of some endemic or subendemic species. Designations: orange ball (*Hyleoglomeris awchasica*), red ball (*Nopoiulus golovatchi*), green triangle (*Cylindroiulus pterophylacum*), red ring (*Cylindroiulus ruber*), white ball (*Polydesmus abchasius*), blue ball (*Trachysphaera fragilis*), green star (*Trachysphaera radiosa*), pink ring (*Cylindroiulus schestoperovi*), yellow ring (*Hirudicryptus abchasicus*).

**Figure 8.**
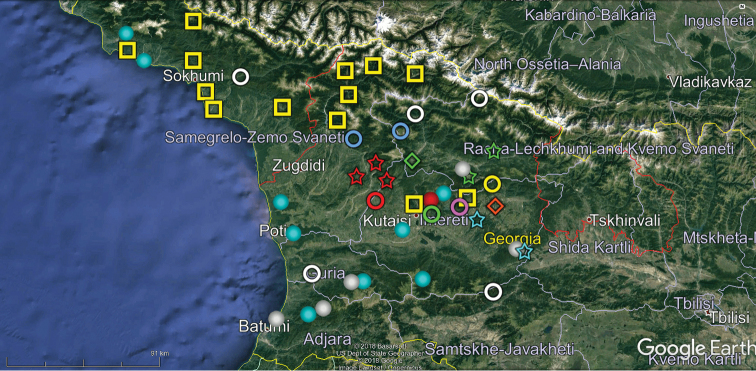
Map of western Georgia (= Colchis) showing the distributions of some other endemic or subendemic species. Designations: green diamond (*Georgiosoma bicornutum*), white ball (*Trachysphaera minuta*), orange diamond (*Trachysphaera orientalis*), red ball (*Trachysphaera solida*), red star (*Typhloglomeris palatovi*), red ring (*Paranotosoma cordatum*), blue star (*Cylindroiulus quadrus*), blue ball (*Cylindroiulus placidus*) , yellow square (*Pachyiulus krivolutskyi*), green star (*Acanthophorella barjadzei*), pink ring (*Metamastigophorophyllon lamellohirsutum*), blue ring (*Paranotosoma attemsi*), yellow ring (*Ratcheuma excorne*), white ring (*Nemasoma caucasicum*), green ring (*Leucogeorgia longipes*).

**Figure 9.**
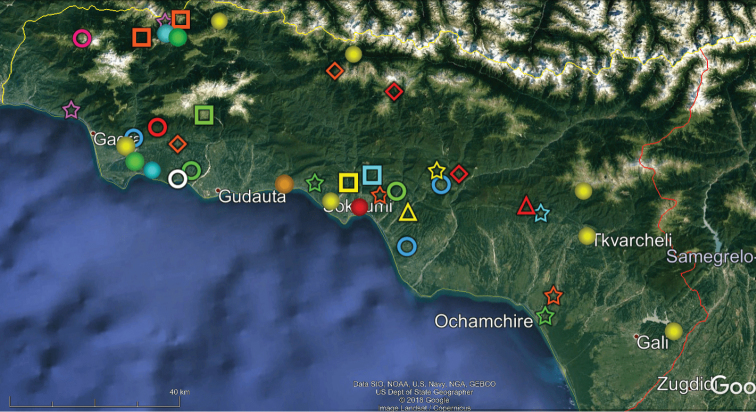
Map of Abkhazia showing the distributions of some endemic or subendemic species. Designations: red triangle (*Brachychaetosoma turbanovi*), blue square (*Caucaseuma kelasuri*), orange star (*Archileucogeorgia abchasica*), pink star (*Omobrachyiulus implicitus*), orange square (*Cylindroiulus olgainna*), blue ring (*Paranotosoma subrotundatum*), yellow star (*Julus lindholmi*), green star (*Leucogeorgia rediviva*), green ring (*Dentatosoma magnum*), pink ring (*Heterocaucaseuma deprofundum*), orange diamond (*Metamastigophorophyllon giljarovi*), white ring (*Kubaniulus gracilis*), blue star (*Metamastigophorophyllon hamatum*), red ring (*Pseudoflagellophorella mirabilis*), red diamond (*Megaphyllum dioscoriadis*), yellow square (*Nopoiulus brevipilosus*), yellow triangle (*Archileucogeorgia satunini*), orange ball (*Heterocaucaseuma longicorne*), red ball (*Omobrachyiulus hortensis*), blue ball (*Brachydesmus furcatus*), green ball (*Brachydesmus simplex*), yellow ball (*Polydesmus lignaui*), green square (*Heterocaucaseuma mauriesi*).

**Figure 10.**
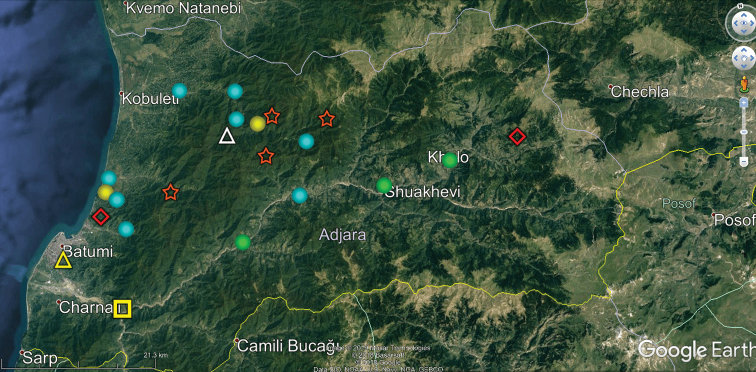
Map of Ajaria showing the distributions of some endemic or subendemic species. Designations: blue ball (*Adshardicus strasseri*), red diamond (*Brachydesmus kvavadzei*), green ball (*Dentatosoma denticulatum*), orange star (*Dentatosoma zeraboseli*), yellow square (*Omobrachyiulus adsharicus*), white triangle (*Omobrachyiulus divaricatus*), yellow ball (*Paranotosoma cordatum*), yellow triangle (*Syrioiulus adsharicus*).

**Figure 11.**
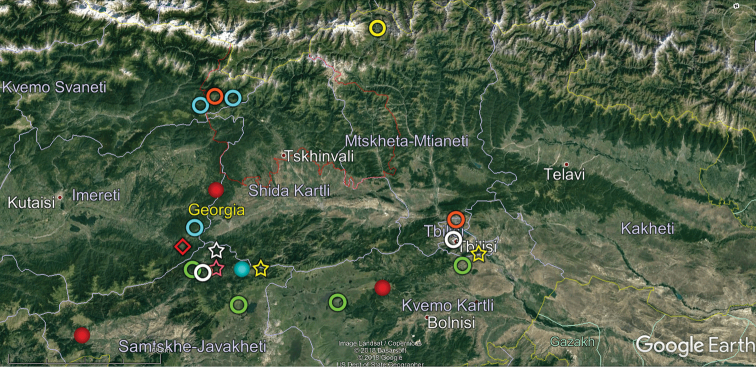
Map of central Georgia showing the distributions of some endemic or subendemic species. Designations: blue ring (*Brachydesmus kalischewskyi*), yellow ring (*Caucaseuma variable*), green Ring (*Catamicrophyllum caucasicum*), red ball (*Cylindroiulus crassiphylacum*), orange ring (*Cylindroiulus pterophylacum*), white ring (*Grusiniulus redikorzevi*), yellow ball (*Herculina oligosagittae*), blue ball (*Herculina polysagittae*), pink star (*Leptoiulus hastatus*), red diamond (*Metamastigophorophyllon martensi*), yellow star (*Omobrachyiulus macrourus* (= *O. m. abchasicus*)), white star (*Syrioiulus georgicus*).

**Figure 12.**
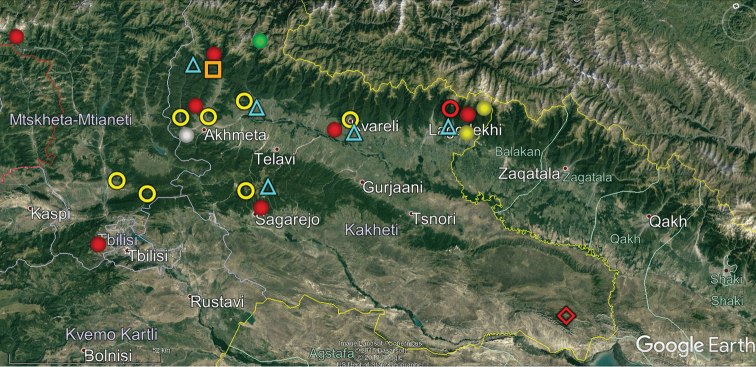
Map of eastern Georgia showing the distributions of some endemic or subendemic species. Designations: green ball (*Alpinella waltheri*), red ball (*Brachydesmus assimilis*), blue triangle (*Cylindroiulus kacheticus*), yellow ball (*Cylindroiulus parvus*), yellow ring (*Hyleoglomeris specialis*), orange square (*Julus kubanus*), red diamond (*Leptoiulus tanymorphus*), white ball (*Pseudoflagellophorella eskovi*), red ring (*Pseudoflagellophorella papilioformis*).

This picture is hardly surprising, as due to the proximity to the Black Sea the climate of western Georgia is largely humid warm temperate. More easterly, the climate is increasingly dry and hot, already dominating eastern Georgia (Bondyrev et al. 2015). Following this trend, the millipede fauna is increasingly depauperate: at least 79 diplopod species occur in western Georgia (= Colchis), but this number drops down to 37 in the central and to 25 in the eastern parts of Georgia (Table 1, Figs 7–12). Millipedes are mainly confined to forests in the Caucasus and in Georgia reflecting their terrestrial, meso- to hygrophilous, largely also calciphilous, arthropod relationships which are historically, trophically and ecologically closely associated with forested biomes (Golovatch and Kime 2009). Dry steppes and arid light forests in central and eastern Georgia (Table 2), as well as the Colchidan swamps of western Georgia support only very few millipede species. Especially tolerant to xeric conditions seems to be *Leptoiulus tanymorphus* (Attems, 1901) (Fig. 12), whereas both *Hirudisoma roseum* (Victor, 1839) and *Julus colchicus* Lohmander, 1936 (Fig. 6), as well as several Chordeumatida tend to represent particularly hydrophilous epigean species. Nearly all cavernicoles (e.g., *Leucogeorgia* spp.) are likewise highly hydrophilous.

As noted above, due to the quite extensive karsts that blanket much of western Georgia, in particular Abkhazia, Samegrelo, Racha Lechkhumi and Imereti, a large proportion of the total fauna is taken up by true cavernicoles (14 species, or 13%). The bulk, however, remains forest-dwelling millipedes and their woody habitats mainly are more or less montane. Present-day Georgia enjoys a remarkable network of nature reserves and national parks, with more than 1/3 of the entire national territory still covered with mountain forests. In contrast, its lowland woodlands have largely been destroyed and long replaced by agri- or sylvicultures, as well as orchards and vineyards (https://apa.gov.ge/en/protected-areas/national-park).

Following Gulisashvili (1964) and Nakhutsrishvili (2013), the altitudinal nature zonation of Georgia can crudely be presented in a tabular form (Table 2). The zonation varies quite clearly in different parts of Georgia (Fig. 1) in relation to climatic gradients. Central Georgia (Figs 1, 4), which is climatically closer to the eastern part of the country, warrants recognition as a separate entity based at least on the distribution of several endemic or subendemic species of Diplopoda (Fig. 11).

No transects are contained in Figures 6–12 to avoid an “overcrowded” presentation of the numerous species distributions; however, these are easy to extrapolate from the figures and thus to follow the general trends and variations in the macro relief of the corresponding parts of Georgia. Only relatively few millipedes occur in subalpine to alpine environments (usually ≥ 2200 m a.s.l.) in Georgia (Table 2). Yet nearly none of them can be considered as being characteristic of the high altitudes, because the same species appear to populate lower elevations as well, down to almost sea-level: *Caucaseuma variabile* Antić & Makarov, 2016, *Dentatosoma magnum* Antić & Makarov, 2016, *Metamastigophorophyllon hamatum* Antić & Makarov, 2016, *Chaetoleptophyllum flexum* Golovatch, 1979, *Cylindroiulus placidus* (Lignau, 1903), *Strongylosoma kordylamythrum* Attems, 1898, *Brachydesmus assimilis* Lohmander, 1936, *B. kalischewskyi* Lignau, 1915, *Polydesmus abchasius* Attems, 1899 and *P. lignaui* Lohmander, 1936. The same concerns *Omobrachyiulus brachyurus* (Attems, 1899) and *Catamicrophyllum caucasicum* (Attems, 1901), both of which occur also at ≤ 2500 m a.s.l. in the Caucasus Minor of Armenia and Azerbaijan; the former species also in Dagestan, Russia, Caucasus Major (personal observations). *Nopoiulus kochii* (Gervais, 1847) is a subcosmpolitan species, common also throughout the Caucasus (10–2200 m a.s.l., Table 1), but because the entire genus *Nopoiulus* is particularly diverse in the Caucasus region, the latter could well have also been the origin centre of *N. kochii* (Golovatch and Enghoff 1990).

At the present, the only exception that may possibly be referred to as a high-montane element in the fauna of Georgia, as well as the entire Caucasus, seems to be *Alpinella waltheri* Antić & Makarov, 2016 (2860 m a.s.l., Table 1, Map 12). Even though some species of *Julus*, i.e., *Julus colchicus* Lohmander, 1936 (20–2850 m a.s.l.), *J. kubanus* Lohmander, 1936 (300–2100 m a.s.l.) and *J. lindholmi* Lohmander, 1936 (450–2200 m a.s.l., Table 1, Figs 9, 12), mostly occur over a wide range of altitudes, *J. lignaui* Verhoeff, 1910 (1500–2800 m a.s.l.) is perhaps the sole congener that seems to be inclined to dwelling in high-mountain environments. However, the paucity or even absence of unequivocally high-mountain elements in the Caucasus generally, and in Georgia in particular, requires confirmation, as our knowledge of the millipede fauna of the regions concerned is still far from complete.

**Table 2.** Vertical zonation of Georgia’s vegetation belts.

Vegetation belts	Western Georgia, altitude (m a.s.l.)	Eastern Georgia, altitude (m a.s.l.)
deserts, dry steppes and arid light forests		150–600
forests	0–1900	600–1900
subalpine	1900–2500	1900–2500
alpine	2500–3100	2500–3000
subnival and nival	3100–3600 and > 3600	3000–3500 and > 3500

## Conclusion

Ongoing research on the diplopod fauna of Georgia will undoubtedly reveal many more species and refine their distributions. This particularly concerns several genera of Julidae, including new cavernicolous and epigean ones (D. Antić, A. Evsyukov, B. Vagalinski, personal communications). As a result, the present paper must only be taken as provisional, marking the present state of the art and is certain to be updated in the near future.
